# Smokeless Tobacco Extract (STE)-Induced Toxicity in Mammalian Cells is Mediated by the Disruption of Cellular Microtubule Network: A Key Mechanism of Cytotoxicity

**DOI:** 10.1371/journal.pone.0068224

**Published:** 2013-07-11

**Authors:** Amlan Das, Abhijit Bhattacharya, Subhendu Chakrabarty, Arnab Ganguli, Gopal Chakrabarti

**Affiliations:** Department of Biotechnology and Dr. B.C. Guha Centre for Genetic Engineering and Biotechnology, University of Calcutta, WB, India; Innsbruck Medical University, Austria

## Abstract

Smokeless tobacco usage is a growing public health problem worldwide. The molecular mechanism(s) underlying smokeless tobacco associated tissue damage remain largely unidentified. In the present study we have tried to explore the effects of aqueous extract of smokeless tobacco (STE) on tubulin-microtubule, the major cytoskeleton protein that maintains cells morphology and participates in cell division. Exposure to STE resulted in dose-dependent cytotoxicity in a variety of mammalian transformed cell lines such as human lung epithelial cells A549, human liver epithelial cells HepG2, and mouse squamous epithelial cells HCC7, as well as non-tumorogenic human peripheral blood mononuclear cells PBMC. Cellular morphology of STE-treated cells was altered and the associated disruption of microtubule network indicates that STE targets tubulin-microtubule system in both cell lines. Furthermore it was also observed that STE-treatment resulted in the selective degradation of cellular tubulin, whereas actin remains unaltered. *In vitro*, polymerization of purified tubulin was inhibited by STE with the IC_50_ value∼150 µg/ml and this is associated with the loss of reactive cysteine residues of tubulin. Application of thiol-based antioxidant N-acetyl cysteine (NAC) significantly abrogates STE-mediated microtubule damage and associated cytotoxicity in both A549 and HepG2 cells. These results suggest that microtubule damage is one of the key mechanisms of STE-induced cytotoxity in mammalian cells.

## Introduction

Consumption of smokeless tobacco (ST) as “spit tobacco” or “chewing tobacco” has become a world wide concern for human health due to its increasing adverse effects. Compared to the western world, usage of ST was reported to be more prevalent in South Asian countries [1], although recent studies had revealed the world wide usage of ST-related products [2], [3]. Chewing tobacco, which is predominantly used in India and also in USA, is mixed with betel leaves, areca nut, lime, and catechu and is sold as legally commercial products termed “gutkha.” [4]. The placement of ST in mouth, either snuff or chewing tobacco, is known to induce wrinkled changes in the oral mucosa, associated with oral injury and inflammation and may lead to Snuff dipper’s lesion, also referred to as leukoplakia. It is characterized by an increased prevalence of gingival recession with associated attachment loss, cervical abrasion, and damage of the oral tissues [1]. Usage of ST may also play a contributory role in the development of cardiovascular disease, peripheral vascular disease, hypertension, peptic ulcers, and fetal morbidity and mortality [5]. Compared to the non users, increased incidence of cardiovascular, renal, and respiratory diseases have been observed in ST-users, as revealed by epidemic studies [6].

Smokeless tobacco consists of huge number of toxicants and carcinogens, which are responsible for its adverse health effects [7], [8]. The tobacco-specific nitrosamines (TSNA) are considered to be the most potent among 28 known carcinogens in smokeless tobacco (National Cancer Institute, 1992), due to its strong carcinogenicity [8], [9]. Beside the toxic chemicals like polycyclic aromatic hydrocarbons, nitrite, nitrate, nicotine, formaldehyde, acetaldehyde, acrolein, crotonaldehyde etc are also reported to be present in the smokeless tobacco [10], [11]. Their have been several reports regarding STE-induced cytotoxicity [12]–[14], but the exact molecular mechanism is still uncertain. Exposure to STE is known to cause cell death and apoptosis in several cultured cell lines including oral keratinocytes [12], [15], and macrophages [16].

Previously we have demonstrated that tubulin, the major cytoskeleton protein participating in diverse cellular functions, acts as a potential target for various cytotoxic agents [17]–[19]. Tubulin dimers polymerize to form microtubules, which further mediate cellular processes, such as cell signaling, cell motility, organelle transport, and maintenance of cell polarity, separation of the duplicated centrosomes, and in cell division [20]–[23]. It is reported that tubulin acts a potential target for the oxidative damage in the pathogenesis of several neurodegenerative diseases including Alzheimer’s disease (AD) [24] and Parkinson’s disease [25], Atherosclerosis [26] and lung emphysema [17]. Tubulin heterodimers as well as cellular microtubules were also found to be potential targets for various cytotoxic agents like rotenone [27], 1,4 benzoquinone [18], acenapthenequinone [19], peroxynitrite [28], and cigarette smoke [17], [26], [29], which finally lead to the cellular apoptosis. Presence of 20 reactive cysteine residues in tubulin, makes the protein more susceptible to oxidation or chemical modification [17], [28] and this leads to the proteosomal degradation of the protein [30]. In our previous reports, we have shown that aqueous extract of cigarette smoke (AECS) and parabenzoquinone (PBQ), the major component of cigarette smoke, induced microtubule disruption and apoptosis in lung epithelial by targeting tubulin-sulfhydrils [17], [18], but the other cytoskeleton protein actin remained unaffected.

The aqueous extract of ST formed with the saliva after consumption is not only absorbed locally but also ingested and enters into the systemic circulation. It has been reported that oral administration of the aqueous extract of smokeless tobacco to male rats, resulted in the apoptosis and damage of lung, liver and kidney tissues, along with the significant up regulation of pro-apoptotic and inflammatory genes [14], [31]. Since liver acts as the main site of metabolism of any foreign substance, the extent of exposure and chances of damages are quite high for the hepatic tissues. Thus the precise health effects of ST may not necessarily be limited to oral tissue injury, but rather may induce a systemic toxicity, when taken for a considerable long period of time**.** A major systemic catastrophe mediated by STE includes damages of hepatic and lung structural and functional devices [31]. Thus in the present study we have tried to delineate a common mechanism of STE-induced cellular toxicity. To visualize the larger spectrum of the cytotoxicity mechanism(s), we have selected human liver epithelium cells (HepG2) and human lung epithelium cells (A549) as *in vitro* models. Since the cell lines used are transformed in nature and may not exactly mimic the normal physiological condition, to assess the cytotoxicity of STE on normal cells, we investigated the cytotoxic effects of STE on a non-tumorigenic cell line PBMC (human peripheral blood mononuclear cells). It has been reported that, STE-treatment resulted in the generation of ROS in mammalian cells [12], [13]. The other probable mechanisms of cytotoxicity were investigated in the present study. Since tubulin-microtubule acts as a potential target for various cytotoxic agents, the intracellular status of microtubules in the absence and presence of different concentrations of STE were examined with both A549 and HepG2 cell lines. Beside the direct effect STE on purified tubulin was also investigated.

## Materials and Methods

### Materials

Nutrient mixture DMEM (supplemented with L-glutamine and sodium pyruvate), Penicillin- streptomycin, Amphotericin B, Trypsin-Versene (1X) and FBS were purchased from GIBCO-Invitrogen, USA. Guanosine 5′-triphosphate (GTP), PIPES, MgCl_2,_ EGTA 5, 5′-dithiobis (2-nitrobenzoic acid) (DTNB), and FITC-conjugated monoclonal anti α-Tubulin antibody (raised in mouse), were purchased from SIGMA, USA. Hepatocellular carcinoma (HepG2) and Lung adenocarcinoma (A549) cells were obtained from National Centre for Cell Sciences, Pune, India. Mouse oral squamous epithelium carcinoma cell line was generous gift from Dr Bipul K Acharya, Weill Cornell Medical College, Cornell University, New York, USA. Bradford protein estimation kit was purchased from GeNei, India. N acetyl cysteine (NAC) was purchased from Sigma and it was dissolved in Phosphate buffer Saline (PBS) pH 7.4. All other chemicals and reagents were purchased from Sisco Research Laboratories, India.

### Preparation of Aqueous Extract of Smokeless Tobacco (STE) Solution

Aqueous extract of smokeless tobacco (khaini) (STE) was prepared as described by Mitchell et al., in [13], with certain modifications. Briefly, 50 ml PBS buffer was added to 10 gm of commercially available smokeless tobacco (brand name Raja Khaini, one of the top selling brands in India), and the mixture was incubated for 24 h at 37°C. It was then filtered first through Whatman filter paper, and subsequently through a 0.22 µ membrane filter paper in sterile condition and pH is adjusted to 7 using 1 M NaOH. The sterile filtrate was then lyophilized to the powdered form. Fresh stocks of STE were prepared from that lyophilized powder in sterile PBS as per experimental requirement.

### Cell Culture and Treatment

Lung epithelial cells (A549), hepatic epithelial cells (HepG2), and mouse squamous epithelial cells (HCC7) were seeded onto plastic tissue culture flasks in DMEM medium containing 200 mg/100 ml Na_2_HCO_3_, 5% fetal bovine serum (FBS), 2 mM L-glutamine, 100 IU penicillin, and 100 mg/ml streptomycin, and incubated at 37°C in a 5% CO_2_-air humidified atmosphere. Human blood peripheral mononuclear cells (PBMC ) were immediately separated by density gradient centrifugation. Briefly, 5 mL blood was layered carefully over equalvolume of Histopaque 1077 and subjected to centrifugation for 30 min at 400×g. PBMC were collected from the buffy layer formed at the plasma–Histopaque 1077 interface and then suspended at a cell count of 1×106 cells/mL in RPMI media. At >80% confluence, cells were washed with PBS, and trypsinized to distribute 1×10^6^ cells/ml in 35 mm plates, which were then treated with different doses of STE for 24 h. To determine the preventive measurement of NAC against STE-mediated toxicity the cells were pre-incubated with 500 µM NAC for 12 h, the media was then decanted and fresh media was added before adding the STE.

### Cell Viability Assay

Cell viability was determined by MTT assay. Cultured mammalian cells were seeded in 96-well plates at 1×10^4^ cells per well, and was allowed to grow to 70%∼80% confluency, and treated with different doses of STE (0–1000 µg/ml) for 48 h. Treated cells were incubated with MTT for 4 h at 37°C, the medium was removed, and dye crystal formazan were solubilized in 150 µl dimethyl sulphoxide (DMSO). Absorbance was measured at 570 nm. Data were calculated as the percentage of inhibition by the following formula:
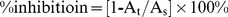
(1)


A_t_ and A_s_ indicated the absorbance of the test sample and solvent control, respectively [32].

### Determination of Apoptotic Population by Annexin V-FITC/PI Double Staining Method

Cultured mammalian cells were treated with the respective IC50 doses of STE and apoptosis was determined by annexinV-FITC/PI (propidium iodide) method. Varying STE doses were employed to HepG2 cells (0 to 400 µg/ml), A549 cells (0 to 300 µg/ml), HCC7 (0 to 400 µg/ml) and PBMC (0 to 300 µg/ml) for 48 h. After decanting the media, live cells were incubated with annexinV-FITC in the binding buffer and then counterstained with propidium iodide (PI). Results were obtained on FACS calibur (Becton Dickinson) using Cell Quest software [33].

### Detection of Mitochondrial Membrane Potential (MMP)

Cultured HepG2 and A549 were grown to a density of 1×10^6^ cells/ml and incubated 24 h in presence STE of different doses (0–300 µg/ml for A549 and 0–400 µg/ml for HepG2). Changes in the mitochondrial membrane potential (MMP) were scrutinized with the fluorescent tagged rhodamine 123 by FACS calibur (Becton Dickinson) using Cell Quest software [32].

### Measurement of Caspase-3 Activity

Caspase-3 activities were assayed in both A549 and HepG2 cells by following the method described in [18]. In this assay, FAM-DEVD-FMK, a cell permeable carboxyfluorescein-labeled fluoromethyl ketone peptide inhibitor (FLICA) of Caspase-3 was used. Cultured HepG2 and A549 were exposed to different STE doses (0–300 µg/ml for A549 and 0–400 µg/ml for HepG2), for 24 h. The green fluorescence of FLICA in the A549 and HepG2 cells was evaluated by fluorescence spectroscopy by monitoring the fluorescence obtained at 520 nm.

### Confocal Microscopy for Cellular Microtubule Structure

Cultured HepG2 and A549 cells were grown to a density 1×10^6^ cells/ml and incubated with different doses of STE (0–400 µg/ml) for 24 h. After treatment cells were fixed with 2% paraformaldehyde and processed further following the published protocol [18]. HepG2 cells were then incubated with mouse monoclonal anti-tubulin antibody (1∶100), followed by rhodamine conjugated secondary antibody (1∶100), while A549 cells were incubated with FITC-conjugated mouse monoclonal anti-tubulin antibody (1∶100). Images of the cellular microtubules were taken by Ziess confocal microscope (LSM 510 Meta).

### Western Blotting of Tubulin and Actin Following STE Treatment

Cultured HepG2 and A549 cells were grown to a density 1×10^6^ cells/ml and treated with different doses of STE (0–300 µg/ml for A549 and 0–400 µg/ml for HepG2) to estimate the intracellular effect of STE on two well known structural proteins tubulin and β-actin. 50 µg of total protein was loaded in each well during SDS-PAGE, and then western blot was carried on. Mouse monoclonal anti-α-tubulin antibody (1∶5000 dilution, obtained from Sigma, USA) and rabbit monoclonal anti-β-actin (1∶1000 dilution, obtained from Sigma, USA) were used as primary antibodies, and HRP-tagged anti-mouse IgG (raised in goat) and HRP-tagged anti-rabbit IgG (raised in goat) were as secondary antibodies (1∶10000 dillution, purchased from Santacruz, USA).

### Purification of Tubulin from Goat Brain

Tubulin was isolated from goat brain by two cycles of temperature-dependent assembly and disassembly in PEM buffer (50 mM PIPES, 1 mM EGTA, and 0.5 mM MgCl_2_ at pH 6.9), in the presence of 1 mM GTP, followed by two more cycles in 1 M glutamate buffer [34]. The purified tubulin, free of MAPs was checked by 8% SDS-PAGE. Aliquots were flash-frozen in liquid nitrogen and stored at –70°C. The protein concentration was estimated by the method of Bradford [35] using bovine serum albumin as the standard.

### Inhibition of Purified Tubulin Assembly *In Cell Free System* by STE

Tubulin (12 µM) was mixed with different doses of STE in polymerization buffer 1 mM MgSO_4_, 1 mM EGTA, 1 mM GTP, 1.0 M monosodium glutamate, pH 6.8), and the assembly reaction was initiated by incubating the sample at 37°C in the presence of different doses of STE (0–200 µg/ml). The rate and extent of the polymerization reaction were monitored by light scattering at 350 nm.

### Transmission Electron Microscopy (TEM) Study for Detecting Microtubule Polymers

Samples for TEM were prepared following the published protocol [17]. Tubulin (1.2 mg/ml) was polymerized at 37°C in the absence and presence of different doses of STE, for 1 h in a 300 µl mixture. Microtubules were then fixed in 0.5% pre-warmed glutaraldehyde for 5 min. Each sample (10 µl) was loaded in carbon-coated electron microscope grids (300-mesh) for 20 s and blotted dry. The grids were subsequently negatively stained with 1% uranyl acetate and air-dried. The samples were viewed using a Philips Fei Technai G212 electron microscope. Images were taken at 20000× magnifications.

### Measurement of Reactive Cysteine Residues of Tubulin by DTNB Assay

Formation of thio-nitrobenzoate anion (TNB) by DTNB reaction with the free sulfhydryl groups was measured by monitoring absorbance at 412 nm, and number of reactive cysteine residues were calculated using ε_412 = _13,600 M^−1^cm^−1^ for TNB [36]. Tubulin (10 µM) in 50 mM PEM buffer, was incubated with different doses of STE (0 µg/ml 200 µg/ml) in 200 µL final volume, at 37°C for 15 min. After the incubation, tubulin was diluted 10-fold to make the final tubulin concentration 1 µM, and the numbers of reactive cysteine were estimated spectrophotometrically by DTNB kinetics.

### Statistical Analysis of Data

Data are presented as the mean of at least three independent experiments along with standard error of the mean (SEM). Statistical analysis of data was done by one-way analysis of variance (ANOVA), with Student-Newman-Keul test by using Sigma plot 11.0. The p value <0.05 was considered to be statistically significant.

## Results

### Loss of Viability of Mammalian Cell Lines upon Treatment with STE

**Figure 1:**
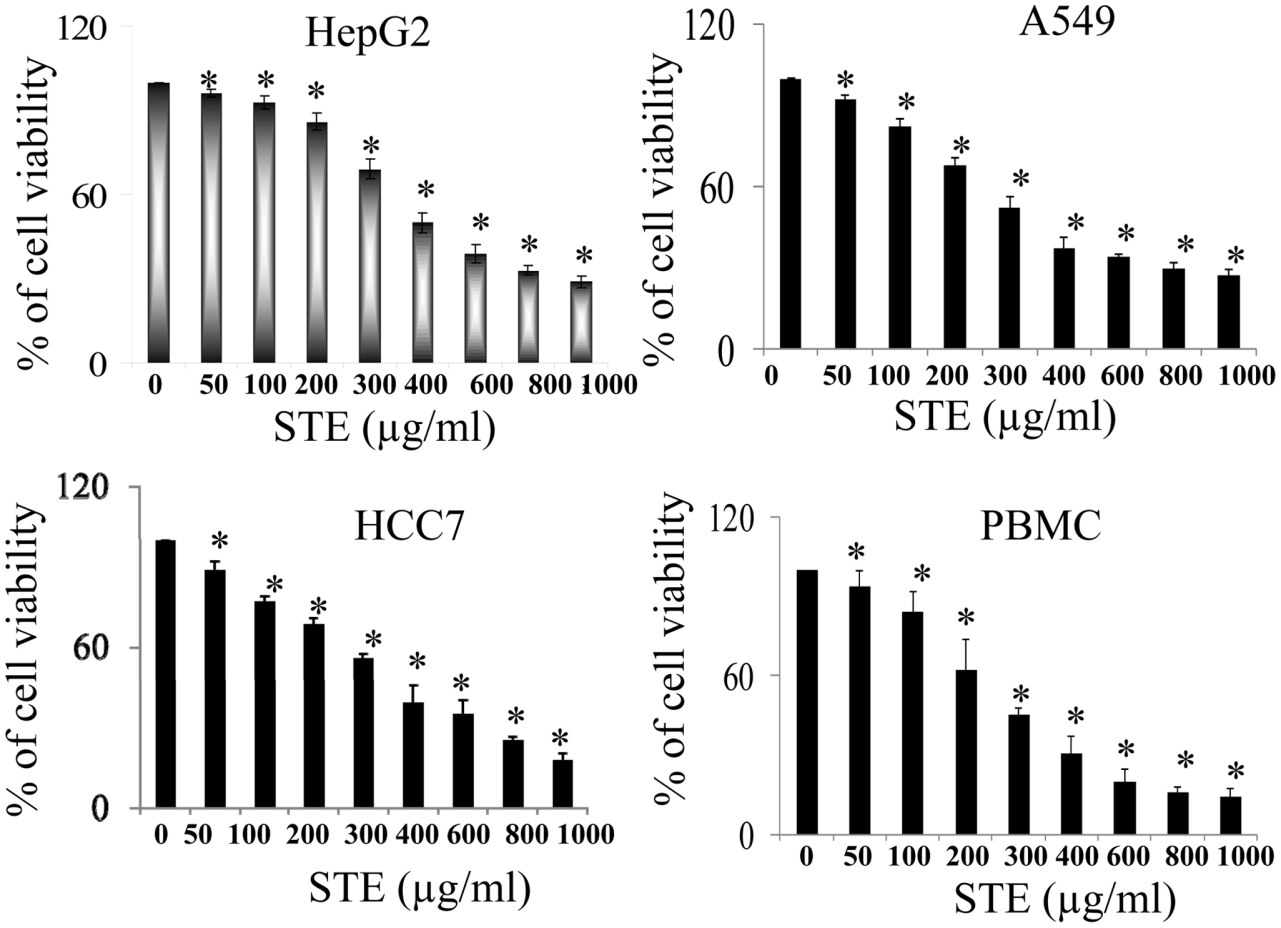
Induction of cytotoxicity in mammalian cells by STE. Cultured mammalian cells were incubated in the absence and presence of different doses of STE (0 to 1000 µg/ml of STE) for 48 h and cell viability was determined by MTT assay. Data are represented as the mean±SEM [*P<0.05 vs control (STE-untreated cell), where n = 4].

Cell viability experiments (MTT assay) were performed using human liver epithelial cells HepG2, lung epithelial cells A549, mouse squamous epithelial cells HCC7 and human peripheral blood mononuclear cells PBMC in the presence of different doses of STE after incubation for 48 h (Fig. 1). A dose-dependent loss of viability was observed, when the cells were treated with STE and the respective IC_50_ observed were around 400 µg/ml for HepG2 cells, 300 µg/ml for A549 cells, 430 µg/ml for HCC7 cells and 300 µg/ml for PBMC.

### Induction of Apoptosis in Mammalian Cell Lines by STE

**Figure 2:**
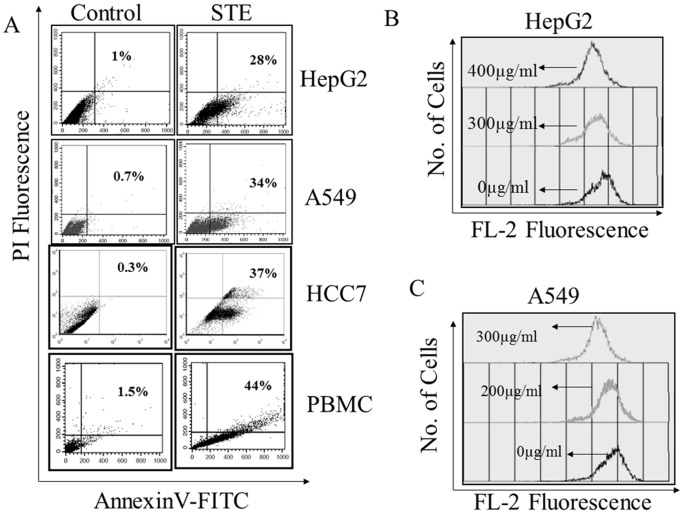
Induction of apoptosis and loss of mitochondrial membrane potential (MMP) in STE- treated cultured cells. (A) Induction of apoptosis in STE-treated mammalian cells was determined by annexin V-FITC and PI double staining. Apoptotic cells were analyzed flow cytometrically, and a dot plot representation of annexin-V-FITC-fluorescence (*x*-axis) vs PI-fluorescence (*y-*axis) has been displayed. Mitochondrial membrane potential in STE-treated HepG2 cells (B) and A549 cells (C) were monitored flow cytometrically by using the fluorescent probe Rhodamine 123. MMP was determined using a FACS Calibur flow cytometer (BD) with excitation at 488 and emission at 535 nm. Results are expressed as a histogram analysis, average of three experiments with SEM [P<0.05 vs control (STE-untreated cell), n = 3].

Smokeless tobacco extract induced apoptosis in mammalian cells were monitored flowcytometrically by FITC-annexin-V/propidium iodide (PI) double staining assay. A significant amount of annexinV positive (early apoptotic) cells were observed, when the cells were treated with STE (Fig. 2A). At the respective IC_50_ dose, apoptotic population in STE-treated cells increased significantly. About 28% of STE-treated HepG2 cells were found to be apoptotic where as in A549 and HCC7 cells, the apoptotic population was found to be 34% and 37%, respectively. When PBMC were treated with STE, about 43% of the cells were found to be apoptotic. These results indicated that exposure to STE a triggers cell death and apoptosis in the cultured mammalian cells.

### Loss of Mitochondrial Membrane Potential (MMP) and Activation of Caspase-3 in the STE- Treated HepG2 and A549 Cells

To examine the involvement of mitochondria in STE-induced apoptosis, alterations in the mitochondrial membrane potential (MMP) were monitored with the fluorescent probe rhodamine 123 by FACS. A gradual decrease in the rhodamine florescence intensity was observed when HepG2 and A549 cells were treated with different STE doses (0–400 µg/ml). For STE-treated HepG2 cells, rhodamine-mean fluorescence intensity (MFI) was found to be decreased from 823 in the untreated cells, to 640 at 300 µg/ml STE dose and 532 at 400 µg/ml STE dose respectively (Fig. 2B). Similarly for A549 cells STE- treatment resulted in the decrease of MFI from 890 in the untreated cells to 750 at 200 µg/ml STE dose and 460 at 300 µg/ml STE dose respectively (Fig. 2C).

**Figure 3:**
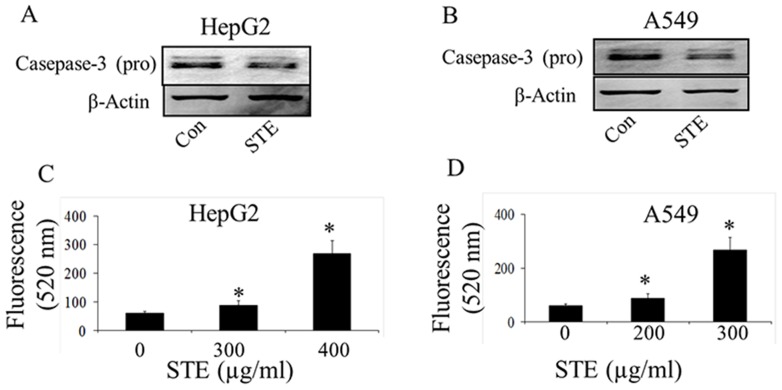
Activation of caspase-3 in STE- treated HepG2 and A549 cells. Activation of caspase -3 in STE-treated HepG2 cells and A549 cells was determined by western blot and fluorescence based assay. Status of pro-caspase-3 in STE (IC_50_ dose)-treated cell lines was determined by western blotting (A & B). Activity of caspase-3 in STE-treated HepG2 cells (C) and A549 cells (D), were determined by measuring the green fluorescence of the carboxyfluorescein-labeled fluoromethyl ketone peptide inhibitor (FLICA), using an excitation wavelength of 490 nm and an emission wavelength of 520 nm by a Jasco F 6300 spectrofluorimeter. Data represent the mean ±SEM (*p<0.05 vs control, *n = *3).

Activation of the caspase-3 is an obvious downstream event in the mitochondrial dependent apoptotic pathway. Activation of the caspase-3 in control and STE-treated HepG2 and A549 cells were assessed by both western blot against pro-caspase-3 and determination of caspase-3 activity by fluorometric analysis (Fig. 3). Expression levels of pro-caspase-3 were reduced significantly in both HepG2 and A549 cells when treated with the respective IC_50_ dose of STE, as confirmed by western blot (Fig. 3A,C). Activation of caspase-3 was confirmed by monitoring the fluorescence obtained at 520 nm, upon excitation at 490 nm (Fig. 3B,D). When HepG2 cells were treated with STE around 1.5±0.1 -fold increase in caspase-3 activity was observed at 300 µg/ml STE dose and on treatment with 400 µg/ml STE, caspase-3 activity was increased by 4.4±0.20 fold (Fig. 3B). A dose-dependent increase in caspase-3 activity was also observed, when A549 cells were treated with STE. Around 2±0.4-fold increase in the caspase-3 activity was observed at 200 µg/ml STE dose and on treatment with 300 µg/ml STE, caspase-3 activity was increased by 5.0±0.2-fold (Fig. 3D).

### Alteration in Cellular Morphology and Inhibition of Migratory Properties of STE-Treated HepG2 and A549 Cells

**Figure 4:**
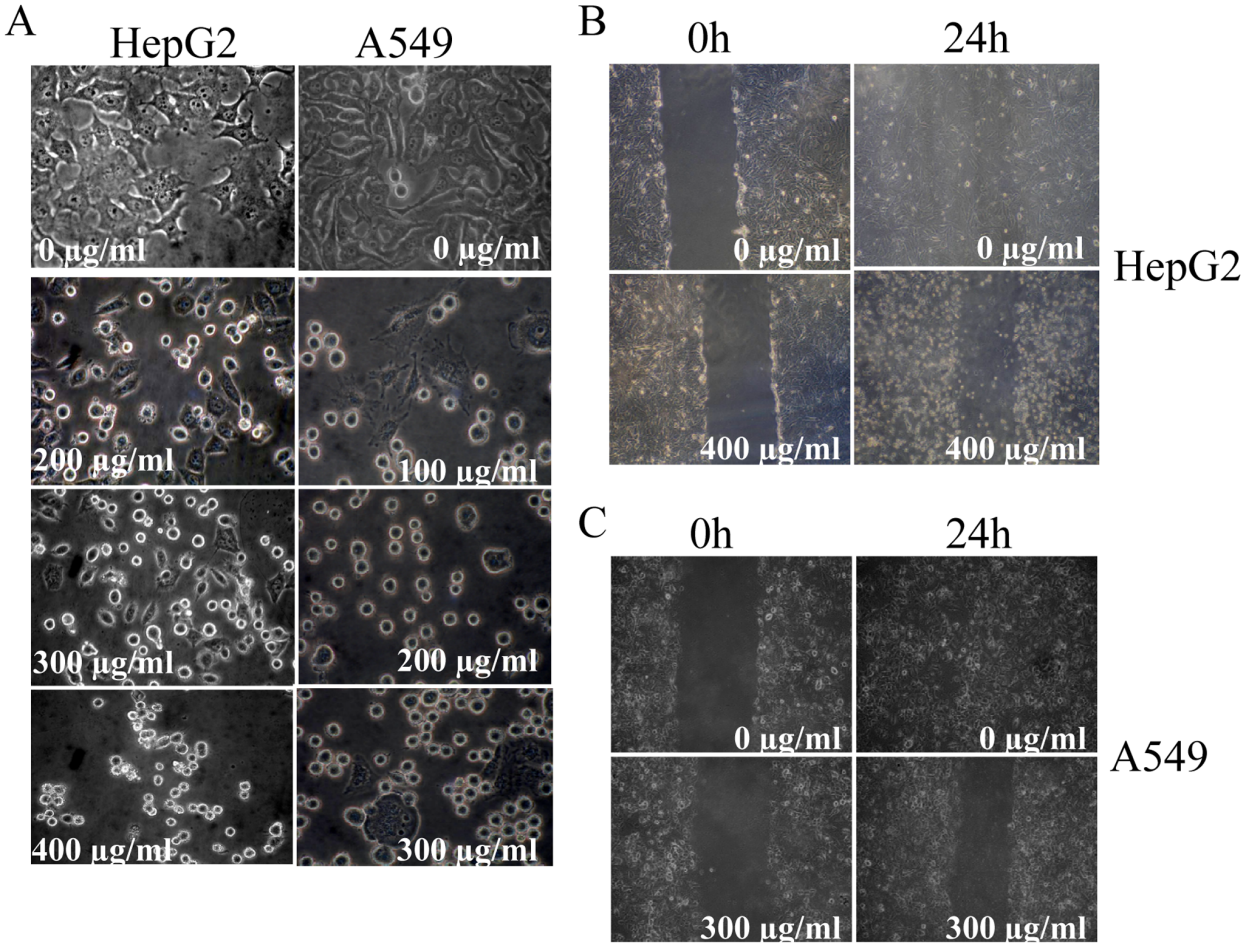
Alteration of cellular morphology and inhibition of cell migration in STE-treated HePG2 and A549 cells. (A) Contraction and shrinkage of cellular morphology of HepG2 and A549 cells in the presence of STE. (B) Inhibition of the migration of HepG2 cells in the absence and presence of 400 µg/ml STE for 24 h. (C) Inhibition of the migration of A549 cells in the absence and presence of 300 µg/ml STE for 24 h.

The normal morphology of the HepG2 and A549 cells was found to be altered by STE in a dose-dependent manner. Cultured HepG2 and A549 cells were treated with various STE doses (0–400 µg/ml), incubated for and 24 h, and phase contrast images of the cells were captured by the Olympus inverted microscope. The untreated cells have regular cellular morphologies, but aberrations in the morphology were observed after the STE treatment, in a dose-dependent fashion (Fig. 4A). With the gradual increase in STE dose, significant shrinkage and contraction of cytoplasmic materials were observed in both HepG2 and A549 cells accompanied by the complete loss of cellular integrity.

Migratory activities of HepG2 and A549 cells in the absence and presence of STE were observed by the wound-healing assay. Confluent monolayers of cultured HepG2 and A549 were scraped with a plastic pipet tip to create a wound, and then incubated with the respective IC_50_ dose 24 h (Fig. 4B,C). The untreated cells were found to heal the wound after 24 h of incubation, but in the presence of STE, the treated cells completely failed to migrate. This observation clearly indicated that STE inhibited the migratory properties of the mammalian cells.

### Irreversible Disruption of Microtubule Network in STE-Treated HepG2 and A549 Cells, Followed by the Degradation of Total Tubulin

**Figure 5:**
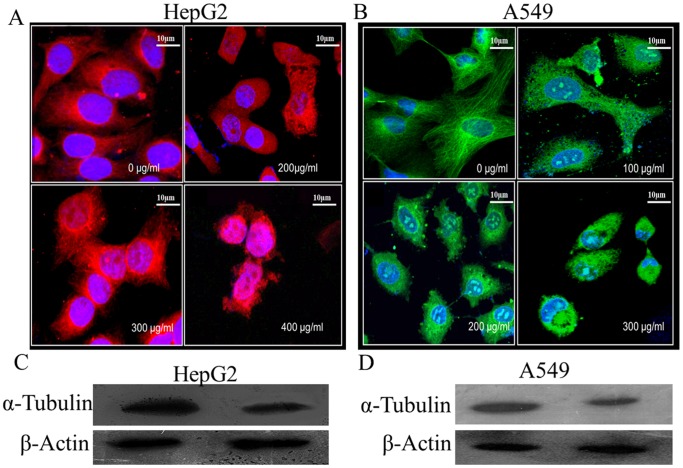
STE-mediated disruption and degradation of microtubules in HepG2 and A549 cells. Cultured HepG2 and A549 cells were treated with different doses of STE (0 to 400 µg/ml of STE). After 24 h treatment, cells were incubated with mouse monoclonal anti-α-tubulin antibody and corresponding rhodamine tagged secondary antibody for HepG2 cells and mouse monoclonal anti-α-tubulin antibody conjugated with FITC. Images of the untreated and STE-treated HepG2 cells (A) and A549 cells (B) were captured by a Ziess confocal microscope, LSM 510 meta. Western blot analysis against tubulin and actin proteins in HepG2 cells (C) and A549 cells (D) treated with STE (0–400 µg/ml) using mouse monoclonal anti-α-tubulin and rabbit monoclonal anti-β-actin antibodies. Data are represented as best of three independent experiments.

Since microtubules play important roles in the maintenance of cellular architecture and migratory activities of mammalian cells, we investigated the status of the cellular microtubules in the STE-treated HepG2 and A549 cells. The organization of microtubule structure in the absence and presence of STE were examined by confocal microscopy (Fig. 5A,B). In case of untreated cells, regular microtubule structures were observed, but with the increasing STE concentrations microtubule disruption took place in both the cell lines. In the untreated HepG2 cells fibrous microtubule structures were observed under the confocal microscope, but at relatively lower STE doses e.g. 200 µg/ml and 300 µg/ml (<IC_50_ dose) microtubule disruptions were very prominent. Further at 400 µg/ml STE concentration microtubule disruption was aggravated (Fig. 5A). Similarly for A549 cells aberration of the normal microtubule structure was initiated at the dose of 100 µg/ml, and at the doses of 200 µg/ml and 300 µg/ml of STE, microtubule structures were totally disrupted (Fig. 5B).

It was reported that chemical modification of tubulin in cells resulted in the proteosomal degradation of the protein [30]. Degradation of tubulin in STE-treated HepG2 cells was observed by western blotting (Fig. 5C). Cultured HepG2 cells, when incubated for 24 h in the presence of 400 µg/ml STE (IC_50_ dose), resulted in tubulin degradation. Under similar experimental conditions, status of another major cytoskeletal protein actin was checked, and degradation of actin was not observed in STE-treated HepG2 cells. Similar result was obtained when A549 cells were treated with STE (400 µg/ml) and western blots for tubulin and actin were performed (Fig. 5D). These results indicated that STE specifically interacts with tubulin in cultured mammalian cells.

**Figure 6:**
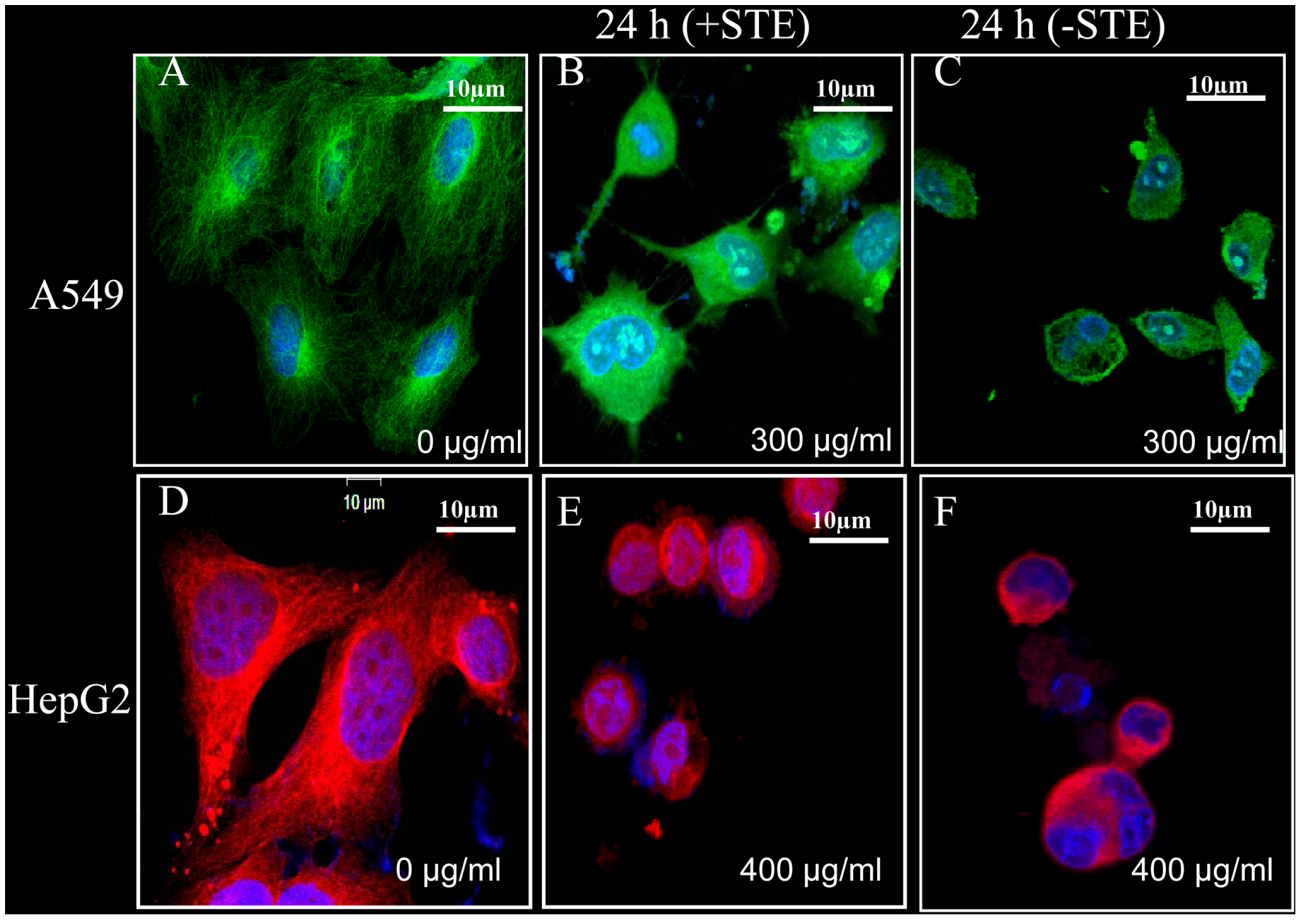
Irreversible disruption of the microtubule network in STE-treated A549 and HepG2 cells. Cultured A549 and HepG2 cells were treated with respective IC_50_ STE doses, with appropriate control sets (untreated cells). After 24 h of incubation, the media containing STE was replaced with fresh normal media without STE as discussed in methods. (A–C) Control and treated A549 cells were incubated with FITC-conjugated mouse monoclonal anti-tubulin antibody and images were captured by a Ziess confocal microscope LSM 510 meta. (D–F) Control and treated HepG2 cells were incubated with anti-tubulin (mouse monoclonal) antibody and corresponding rhodamine conjugated (red) secondary antibody and images were captured by a Ziess confocal microscope LSM 510 meta. The results represent the best of data collected from three experiments with similar results.

Further investigations revealed that disruption of microtubule network in STE-treated HepG2 and A549 cells occurred in an irreversible manner. Cells were treated with the respective IC_50_ dose of STE for 24 h, and the old medium was then replaced with fresh medium without STE and again incubated for 24 h. Confocal images of microtubules in untreated (Fig. 6A,D) and treated (Fig. 6C,F) cells revealed that the damaged microtubules in the STE-treated cells failed to recover.

These results clearly indicated that the disruption of the interphase microtubules in the STE treated cultured cell lines were responsible for the aberration of cellular morphology and inhibition of migratory properties.

### Inhibition of Purified Tubulin Polymerization by STE

**Figure 7:**
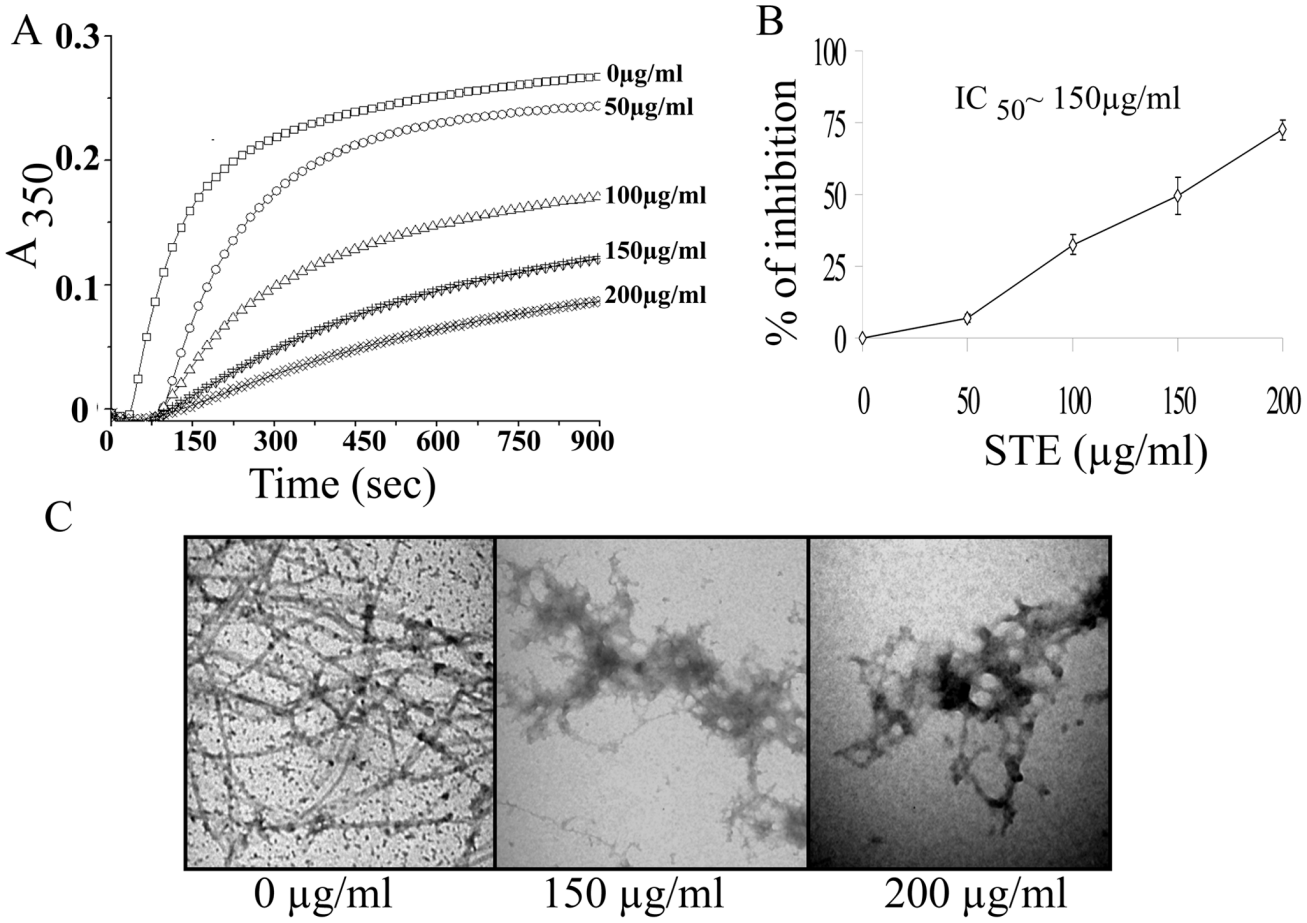
Inhibition of the assembly of purified tubulin by STE. (A) Tubulin assembly study. Tubulin (12 µM) was polymerized separately in the presence of (0 −200 µg/ml) STE at 37°C. The progress of tubulin assembly was monitored spectrophotometrically at 350 nm. (B) A plot of percentage of polymerization inhibition against dose of STE. Data represent the mean ±SEM (*p*<0.05 vs control, *n = *3). (C) Aggregation of microtubule protofilaments in the presence of STE as observed by a transmission electron microscopy. Tubulin (12 µM) was polymerized separately in the presence of different STE doses (0–200 µg/ml), and the images were taken at 20000X magnification. The bar represents 500 nm. The results represent the best of data collected from three experiments with similar results.

As smokeless tobacco disrupted of microtubule network of HepG2 and A549 cells, we like to know whether STE inhibits polymerization of purified tubulin into microtubules. Inhibition of microtubule assembly by STE was studied *in cell-free system* by light scattering experiment by monitoring absorbance at 350 nm. Purified tubulin (12 µM) was polymerized in the absence or presence of different doses of STE as described in the ‘Materials and Methods’. The STE was found to inhibit the rate and extent of tubulin polymerization in a dose-dependent manner (Fig. 7A). The percentage inhibition of microtubule polymerization was calculated using the steady-state absorbance readings in the absence and presence of different doses of STE (Fig. 7B). Around 54% inhibition of tubulin polymerization was occurred at STE dose of 150 µg/ml. Again in a 200 µg/ml dose of STE, 70% inhibition of tubulin polymerization was observed.

This result was further confirmed by transmission electron microscopy study (Fig. 7C). In the untreated set, tubulin dimers polymerize efficiently to form the polymeric microtubules as evident from the micrograph image. But in the presence of STE doses of 150 µg/ml and 200 µg/ml, tubulin aggregates were observed instead of the polymeric mass. These results clearly indicate that STE is interfering with the polymerization properties of tubulin dimers.

### Loss of Reactive Cysteine Residues of Tubulin in the Presence of STE

**Figure 8:**
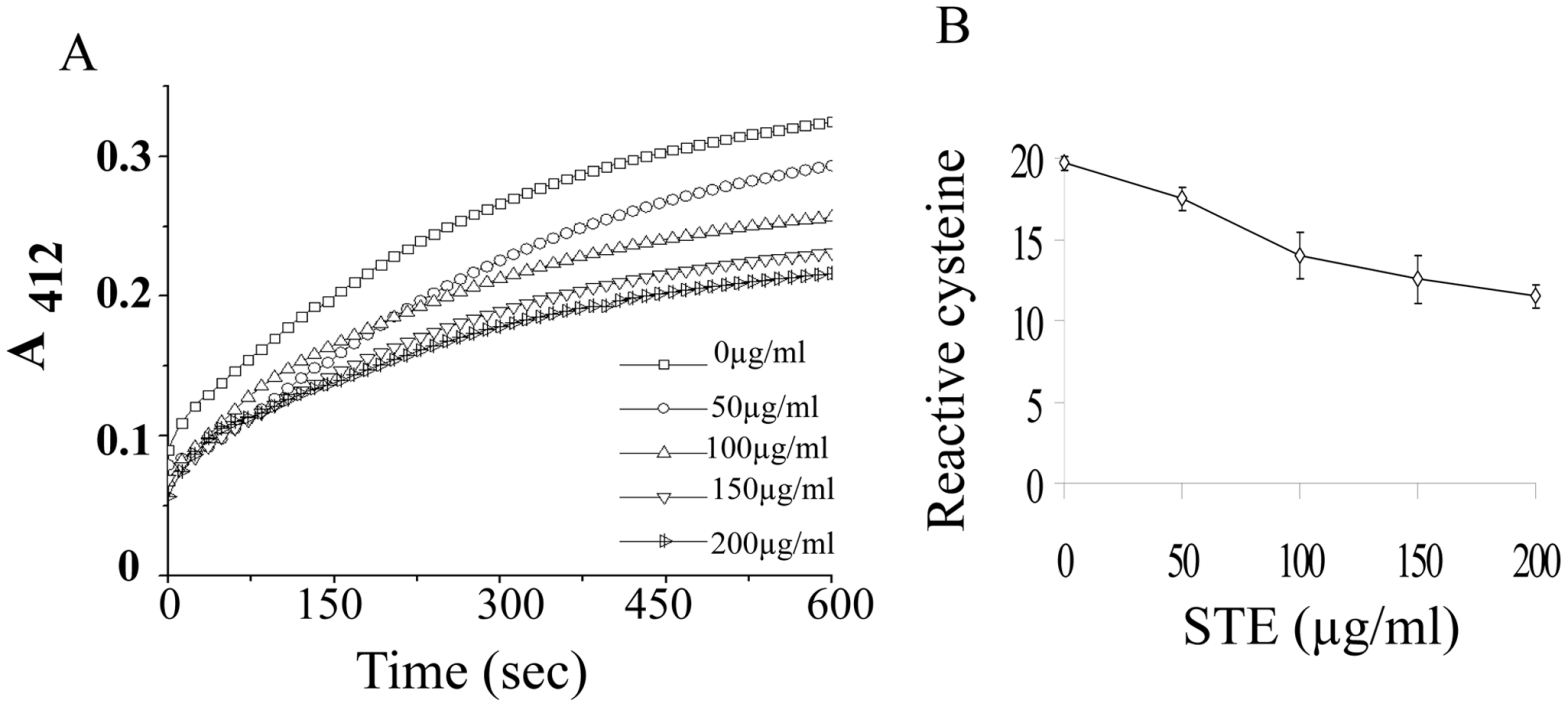
Estimation of reactive cysteine residues of tubulin by DTNB reaction. Tubulin (10 µM) was separately treated with (0 −200) µg/ml STE for 1 h. (A) Samples are diluted for normalization of protein concentration to 1 µM and cysteine residues were estimated by reaction with DTNB as described in the ‘Materials and Methods’. (B) A plot of total available reactive cysteine residues of tubulin against concentration of STE. Data represent the mean ±SEM (*p*<0.05 vs control, *n = *3).

The tubulin dimer has 20 cysteine residues that play important role in folding, and polymerization of tubulin [37], [38] and oxidation or modification of these sulfhydril groups is usually accompanied with the loss of polymerization activity [26], [34]. Among the 20 residues, 18–20 are available for reaction with DTNB [33], and we estimated number of cysteine residues in the absence and presence of STE by DTNB reaction (details in ‘Methods’). A linear decrease in the reactive cysteine residues of tubulin was observed with the gradual increase in STE doses from 0 µg/ml to 200 µg/ml (Fig. 8A,B). In the presence of 150****µg/ml STE, a loss of around 8 cysteine residues was observed as compared with the control.

### N-Acetyl Cysteine (NAC) Mediated Protection of A549 and HepG2 Cells Against STE Mediated Cytotoxicity and Microtubule Disruption

**Figure 9:**
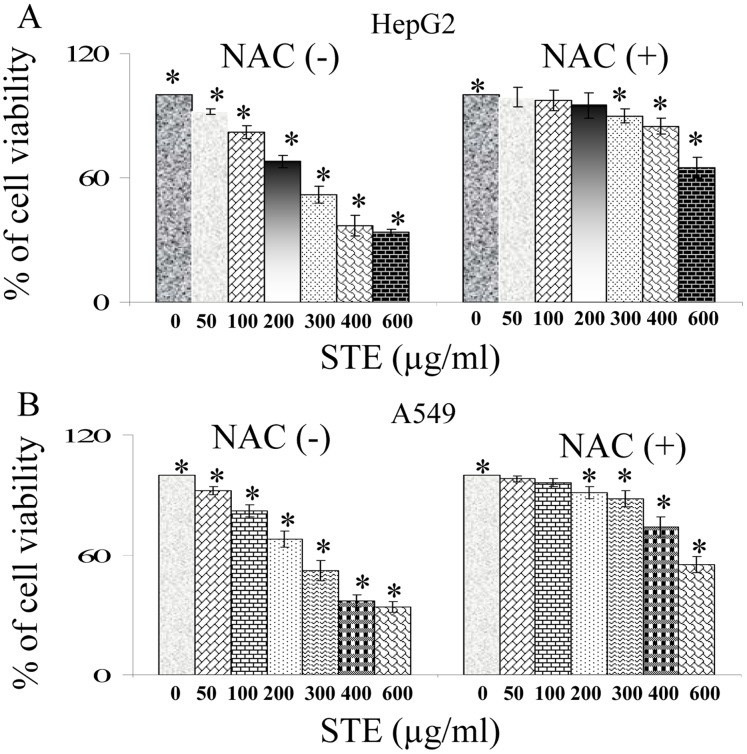
Attenuation of STE induced cytotoxicity in both HepG2 and A549 cells by *N*-acetyl cysteine (NAC). Determination of the viability of HepG2 and A549 cells treated with STE (0–600 µg/ml) alone or incubated with 500 µM NAC prior STE-treatment. Viability for (A) HepG2 and (B) A549 cells was determined by MTT assay. Data are represented as the mean ±SEM (*P<0.05 vs control or STE untreated +NAC treated cells), where n = 4.

N-acetyl cysteine (NAC) is well known and clinically used anti-oxidant which increases cell’s intrinsic anti-oxidant pool. In the current experimental theme, it has been utilized as a protective shield against STE induced cytotoxicity. When HepG2 cells were exposed to STE, the IC_50_ was around 400 µg/ml but on pre-treatment with 500 µM NAC, the cell viability was significantly restored to more than 85% (Fig. 9A). Similarly at STE dose of 300 µg/ml, A549 cells showed 50% viability but upon pre-treatment with 500 µM NAC, cell viability was increased to 88% (Fig. 9B).

**Figure 10:**
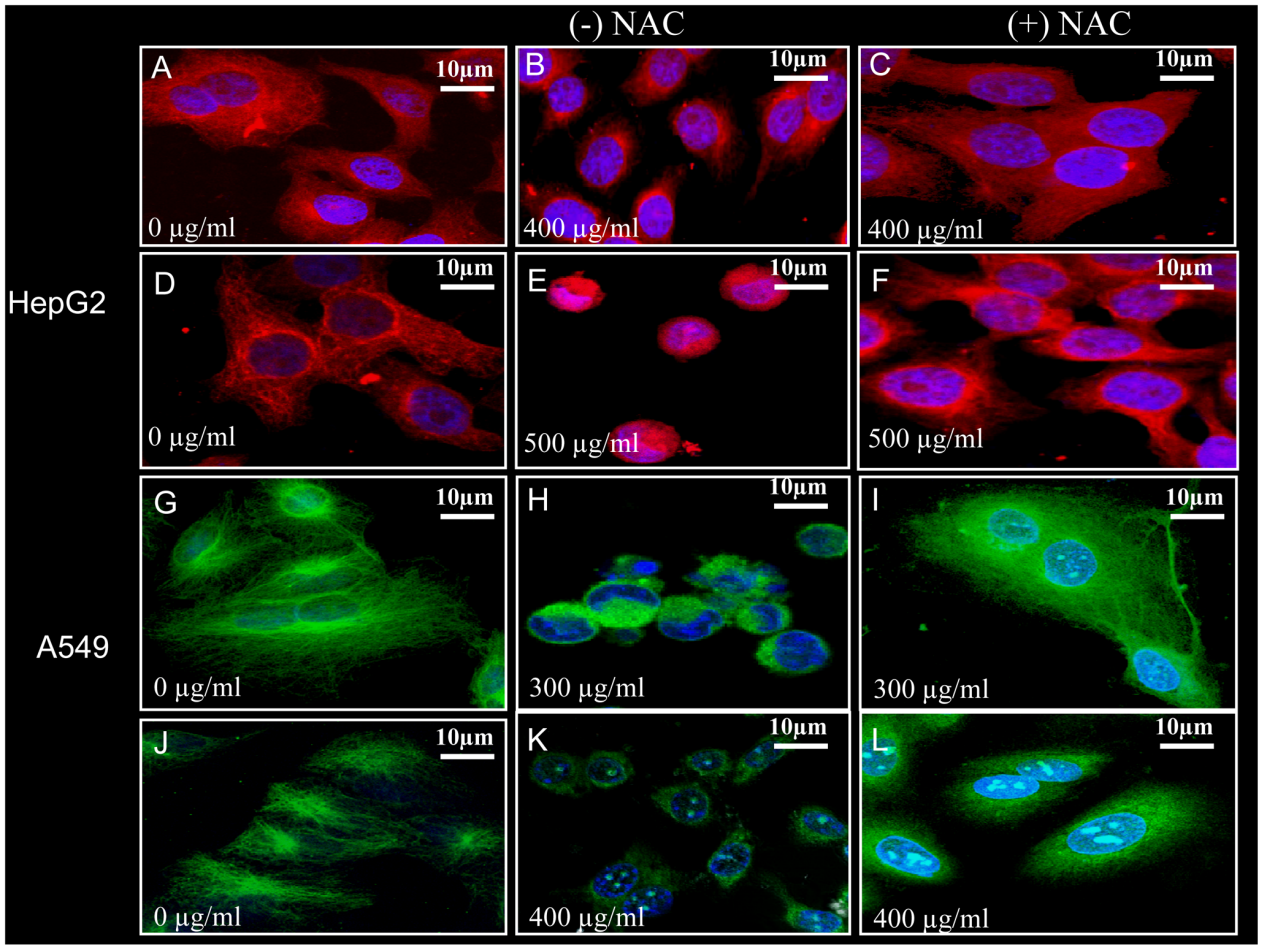
NAC mediated prevention of STE-induced damage of microtubule structure in HepG2 and A549 cells. Microtubules of the HepG2 and A549 cells were probed with mouse monoclonal rhodamine or FITC tagged anti-α-tubulin antibody as before and images were taken by confocal microscope for STE-untreated samples (A,D,J,G), STE-treated samples (B,E,H,K) and NAC-pretreated-then STE-treated samples (C,F,I,L). Details of the experiments are described in ‘Methods’ section. The results represent the best of data collected from three experiments with similar results.

In our previous experiments, we have shown that exposure of HepG2 and A549 cells to STE resulted in the disruption of cellular microtubules. To find out whether NAC acts as a protective parameter, we pre-incubated both HepG2 and A549 cells with 500 µM of NAC for 12 h, before STE treatment (400 and 500 µg/ml). Without pre-treatment with NAC, microtubules were disrupted in the presence of STE (Fig. 10B,E for HepG2 cells and Fig. 10H,K for A549 cells). Very interestingly we observed that NAC pre-treatment is inhibiting STE-induced microtubule disruption in both HepG2 (Fig. 10C,F) and A549 cells (Fig. 10I,L).

## Discussion

Usage of the smokeless tobacco as the “spit tobacco” or “chewing tobacco” in forms of moist smokeless tobacco (MST) or commercially available “Ghutkha”, has become a very common habit worldwide [1]–[3]. Long-term exposure to ST leads to the formation of oral mucosal lesions and tissue injury [1] but the extent of damage was found to be systemic and contributory to the development of cardiovascular disorders [5], and inflammatory responses in lung and hepatic tissues [31]. Smokeless tobacco extract was also known to induce apoptosis and cellular damage [12]–[16], but the precise mechanism is yet unclear. In our previous reports we have demonstrated that microtubules, one of the major cytoskeleton proteins participating in diverse cellular functions, may act as a potential target for tobacco smoke and smoke-components and disruption of the cellular microtubule network leads to apoptosis [17]–[19]. Thus in the present study we have investigated the role tubulin-microtubule in STE-mediated cytotoxicity and apoptosis in mammalian cells.

Application of STE on mammalian cells shows a concentration-dependent decrease in the cell viability as evident from the MTT assay (Fig. 1). Furthermore it was observed that STE-treatment resulted in the induction apoptosis in the treated cell lines and the mitochondrial dependent activation of caspase-3 was also observed (Fig. 2,3). We also observed that induction of apoptosis due to STE-treatment is associated with the loss of cellular architecture and migratory properties of the treated cells (Fig. 4 **)** and further studies revealed that STE-treatment resulted in a gradual perturbation and degradation of the cellular microtubule organization in both HepG2 and A549 cells (Fig. 5) and the effect is dose-dependent and irreversible (Fig. 6). In previous reports we have shown that cigarette smoke extract or smoke component like PBQ selectively targets cellular microtubules but the other house keeping proteins like glyceraldehyde-3-phosphate and β-actin remain unaffected [17], [18]. Similar results were obtained for both HepG2 and A549 cells followed by STE-treatment. It was observed that in both the cell lines, STE-treatment resulted in a drastic decrease in tubulin levels whereas levels of actin remained unaltered. Polymerizing property of the purified tubulin was also inhibited by STE in a dose-dependent fashion (Fig. 7) and this is accompanied by the loss of reactive cysteine residues of tubulin (Fig. 8).

The reactive cysteine residues of tubulin are known to regulate important structural and functional properties of the protein such as folding, and polymerization [37], [38] and any kind of chemical modification of these reactive cysteine residues may result in the proteosomal degradation of tubulin [30]. Thus it may be concluded that tubulin serves as a direct target for STE-components, which may oxidize/modify tubulin sulfhydrils and result in the intracellular degradation of the protein.

In our previous report we have shown that, PBQ, a cytotoxic quinone, present in cigarette smoke and diesel smoke, targets the sulfhydrils of tubulin and induces apoptosis in mammalian cells [18]. Application of the thiolic antioxidant NAC not only reversed PBQ-mediated cytotoxicity, but also conferred protection to the cellular microtubules and purified tubulin against PBQ-mediated damage [18]. Henceforth we have hypothesized that application of NAC improves the intracellular thiol pool directly or indirectly, which in turn inhibits PBQ from targeting tubulin-sulfhydrils. Similar pattern of protection was also observed, when the cells pre-incubated with NAC acquired protection form STE-induced cytotoxicity and STE-mediated microtubule disruption were also inhibited to a significant extent (Fig. 9,10).

Although the experiments have been carried out with the transformed cell lines as *in vitro* models, the novelty of this study is that it aims to find out a specific target for STE-components, which may play an important role in STE-mediated tissue damage. Thus we may conclude that the active components present in STE may target and modify the reactive cysteine residues of tubulin, which subsequently leads to disruption and degradation of cellular microtubules and induces apoptosis in cultured mammalian cells.
