# Correction: Smokeless Tobacco Extract (STE)-Induced Toxicity in Mammalian Cells is Mediated by the Disruption of Cellular Microtubule Network: A Key Mechanism of Cytotoxicity

**DOI:** 10.1371/annotation/704262bd-caf2-4599-ac93-97c7a1ed4601

**Published:** 2014-01-08

**Authors:** Amlan Das, Abhijit Bhattacharya, Subhendu Chakrabarty, Arnab Ganguli, Gopal Chakrabarti

The mouse squamous epithelial cell line HCC7 appears incorrectly throughout the article. The correct cell line is the mouse squamous carcinoma cell line SCC7. 

